# Modulation of Neutrophil Apoptosis and the Resolution of Inflammation through β_2_ Integrins

**DOI:** 10.3389/fimmu.2013.00060

**Published:** 2013-03-06

**Authors:** Driss El Kebir, János G. Filep

**Affiliations:** ^1^Department of Pathology and Cell Biology, University of Montreal and Research Center, Maisonneuve-Rosemont HospitalMontreal, QC, Canada

**Keywords:** neutrophils, Mac-1, apoptosis, lipoxins, resolvins, myeloperoxidase, phagocytosis, resolution of inflammation

## Abstract

Precise control of the neutrophil death program provides a balance between their defense functions and safe clearance, whereas impaired regulation of neutrophil death is thought to contribute to a wide range of inflammatory pathologies. Apoptosis is essential for neutrophil functional shutdown, removal of emigrated neutrophils, and timely resolution of inflammation. Neutrophils receive survival and pro-apoptosis cues from the inflammatory microenvironment and integrate these signals through surface receptors and common downstream mechanisms. Among these receptors are the leukocyte-specific membrane receptors β_2_ integrins that are best known for regulating adhesion and phagocytosis. Accumulating evidence indicate that outside-in signaling through the β_2_ integrin Mac-1 can generate contrasting cues in neutrophils, leading to promotion of their survival or apoptosis. Binding of Mac-1 to its ligands ICAM-1, fibrinogen, or the azurophilic granule enzyme myeloperoxidase suppresses apoptosis, whereas Mac-1-mediated phagocytosis of bacteria evokes apoptotic cell death. Mac-1 signaling is also target for the anti-inflammatory, pro-resolving mediators, including lipoxin A_4_, aspirin-triggered lipoxin A_4_, and resolvin E1. This review focuses on molecular mechanisms underlying Mac-1 regulation of neutrophil apoptosis and highlights recent advances how hierarchy of survival and pro-apoptosis signals can be harnessed to facilitate neutrophil apoptosis and the resolution of inflammation.

## Introduction

Neutrophils form the first line of defense against invading pathogens or tissue injury. They are rapidly recruited to the sites of infection/injury and play a prominent role in the initiation and progression of the inflammatory response. Their many defense mechanisms that destroy invading pathogens are also capable of inflicting damage to the surrounding tissue (Nathan, 2006). Under certain conditions, these harmful consequences became dominant and prolong inflammation. Once the pathogens are cleared, neutrophils are thought to undergo constitutive apoptosis (Savill et al., 2002). This process renders neutrophils unresponsive to extracellular stimuli, allows their recognition and removal by macrophages (Savill et al., 2002; Gilroy et al., 2004; Nathan and Ding, 2010) and limits their potentially harmful actions. Neutrophil accumulation in inflamed tissues is a balance of their recruitment and removal. Conversely, effective resolution of inflammation requires cessation of neutrophil recruitment as well as timely removal of emigrated neutrophils from the site of inflammation. Apoptosis, which ensures that neutrophils are securely marked for disposal, emerged as a control point in resolving inflammation (Filep and El Kebir, 2009; Fox et al., 2010; Perretti, 2012). Pro-survival and pro-apoptosis signals from the inflammatory milieu can, however, influence the execution of the constitutive death program, thereby profoundly affecting the fate of neutrophils and the outcome of the inflammatory response (reviewed in Filep and El Kebir, 2010; Fox et al., 2010; Geering and Simon, 2011).

β_2_ integrins are leukocyte-specific adhesion molecules that govern neutrophil adhesion and transmigration across the activated endothelium and phagocytosis of pathogens (Ross, 2000; Ley et al., 2007). Growing evidence demonstrates that outside-in signaling through β_2_ integrins can generate contrasting cues in neutrophils, leading to promotion of their survival or apoptosis. This review will focus on the hierarchy of these signaling circuits and the underlying molecular mechanisms, and will discuss how interference with β_2_ integrin signaling could be harnessed for promoting neutrophil apoptosis to enhance the resolution of inflammation.

## Neutrophil Apoptosis: A Control Point for the Resolution of Inflammation

### Neutrophil apoptosis during homeostasis and inflammation

Mature neutrophils are terminally differentiated cells that have the shortest lifespan among leukocytes in the circulation. Neutrophil lifespan is generally thought to be in the range of 8–20 h, though recent data with *in vivo* labeling suggest a lifespan of 5.4 days under physiological conditions in humans (Pillay et al., 2010). Aged neutrophils die by constitutive (or spontaneous) apoptosis. This mechanism is essential to maintain the balance of cellular homeostasis under physiological conditions (Cartwright et al., 1964; Coxon et al., 1996). Apoptosis renders neutrophils unresponsive to extracellular stimuli and leads to expression of “eat-me” signals, so that neutrophils can be recognized and removed by macrophages in the spleen and bone marrow and Kupffer cells in the liver (Savill et al., 1989, 2002). In mice, these three organs contribute equally to removal of senescent neutrophils (Furze and Rankin, 2008).

During inflammation, extending the lifespan of neutrophils during transendothelial migration and at the sites of infection is critical for efficient destruction of pathogens (Watson et al., 1997; Savill et al., 2002; Nathan, 2006). Once this is accomplished, neutrophils may undergo necrosis, apoptosis, NETosis (neutrophil extracellular trap cell death) (Brinkmann et al., 2004; Fuchs et al., 2007), or autophagy (Remijsen et al., 2011) with the type of death profoundly affecting the outcome of the inflammatory response.

Apoptotic neutrophil death *in situ* has multiple pro-resolution actions. In addition to becoming unresponsive to agonists and stopping production of inflammatory mediators, apoptotic neutrophils can sequester cytokines (Ariel et al., 2006; Ren et al., 2008) and their phagocytosis by macrophages induces macrophage polarization from a pro-inflammatory (M1) to a pro-resolution (M2) phenotype (Fadok et al., 1998). M2 macrophages secrete mediators, such as IL-10 and TGFβ, which mediate resolution and tissue repair (Ariel and Serhan, 2012; Sica and Mantovani, 2012). Interestingly, injection of large quantities of apoptotic neutrophils protected mice against endotoxin shock (Ren et al., 2008).

In non-resolving inflammation, neutrophils persist at the inflamed site and are liable to cause tissue destruction (Nathan and Ding, 2010; Soehnlein, 2012). Neutrophil recruitment may occur normally or may become excessive, but neutrophils persist as a result of delayed apoptosis or decreased clearance by macrophages (Haslett, 1999; Savill et al., 2002). The abnormal host response creates a persistent inflammatory microenvironment with ongoing release of inflammatory mediators and damage-associated molecular patterns (Nathan and Ding, 2010; Serhan, 2011).

### Neutrophil apoptosis in human disease

The tight regulation of neutrophil death is also evident under pathological conditions, though it is often difficult to decide whether prolonged survival or apoptosis is most favorable from the host’s perspective. Consistently, both accelerated and delayed neutrophil apoptosis could have severe pathological consequences. For example, infections with the opportunistic pathogen *Pseudomonas aeruginosa* (Allen et al., 2005), influenza virus A (Colamussi et al., 1999), or HIV (Elbim et al., 2009) as well as autoimmune diseases, such as systemic lupus erythematosus (Courtney et al., 1999) shorten neutrophil lifespan by accelerating apoptosis, leading to impaired antimicrobial defenses and increased susceptibility to recurrent infections. Intracellular pathogens may use apoptotic neutrophils as a Trojan horse to infect and propagate in macrophages (Laskay et al., 2008; Rupp et al., 2009). On the other hand, delayed neutrophil apoptosis appears to be a component of the pathophysiology in patients with inflammatory diseases, including acute respiratory distress syndrome (ARDS) (Matute-Bello et al., 1997; chronic pulmonary obstructive disease (COPD) (Brown et al., 2009), viral pneumonia (Lindemans et al., 2006), sepsis (Ertel et al., 1998), burn (Chitnis et al., 1996), acute coronary artery disease (Garlichs et al., 2004), rheumatoid arthritis (Wong et al., 2009), and cystic fibrosis (McKeon et al., 2008), and frequently correlates with disease severity and outcome.

### Distinct molecular features of neutrophil apoptosis

A complex network of intracellular death and survival pathways regulates neutrophil apoptosis and the balance of these circuits would ultimately determine the fate of neutrophils. Since neutrophils undergo apoptosis even in the absence of any extracellular stimuli, this type of death is called spontaneous or constitutive programed cell death. However, under most conditions, neutrophils receive both pro-survival and pro-apoptosis cues, and the net effect is likely determined by the balance of these signals. Neutrophil apoptosis shares many morphological features with apoptosis in other cell types; however, it involves distinct molecular mechanisms in executing the cell death program. Predominant expression of the anti-apoptotic protein myeloid cell leukemia-1 (Mcl-1), restricted function of mitochondria to apoptosis, ROS production, release of proteases from azurophilic granules, and unusual roles for nuclear proteins are hallmarks of regulation of apoptosis in neutrophils.

### Mcl-1 regulation of neutrophil survival and apoptosis

Spontaneous neutrophil apoptosis rely upon the balance of pro- and anti-apoptotic members of the Bcl-2 family. Mature human neutrophils constitutively express the pro-apoptotic Bcl-2-associated X protein (Bax), Bcl-2-associated death promoter (Bad), Bcl-2 homologous antagonist/killer (Bak), Bcl-2 homology-3 (BH-3)-interacting domain death agonist (Bid), Bcl-2 interacting protein (Bim), and Bcl-2 interacting killer (Bik) as well as the anti-apoptotic Bcl-2 homolog Mcl-1, and to a much lesser extent A1 and Bcl-X_L_, but not Bcl-2 (Akgul et al., 2001; Moulding et al., 2001). The pro-apoptotic Bcl-2 homologs have relatively long half-lives and their cellular levels change very little during exposure of neutrophils to agents that either accelerate or delay apoptosis. Genetic deletion of *bim* or *bax/bak* results in increased neutrophil numbers in mice (Lindsten et al., 2000). Mcl-1 contains a PEST domain [rich in proline (P), glutamic acid (E), serine (S), and threonine (T)], which facilitates its proteasomal degradation, resulting in a very short half-life (Edwards et al., 2004). Mcl-1 levels closely correlate with neutrophil survival kinetics (Moulding et al., 1989; Hamasaki et al., 1998; Leuenroth et al., 2000; Kato et al., 2006; Dzhagalov et al., 2007). Survival of myeloid cells decreases following treatment with antisense oligonucleotides against Mcl-1 (Moulding et al., 2000). Myeloid lineage-specific knockout of *mcl-1* reduces neutrophil numbers by accelerating apoptosis (Dzhagalov et al., 2007; Steimer et al., 2009). Recent data indicate that Mcl-1 levels drop in advance of apoptosis, even in the presence of caspase inhibitors (Wardle et al., 2011), indicating that Mcl-1 functions as a regulator and a downstream target of caspase activation. On the other hand, unchanged or even increased Mcl-1 expression has been detected in neutrophils of patients with Crohn’s disease (Catarzi et al., 2008) or severe sepsis (Fotouhi-Ardakani et al., 2010). Mcl-1 promotion of neutrophil survival is thought to involve heterodimerization with and neutralization of Bim or Bak in the mitochondrial outer membrane (Reed, 2006; Brenner and Mak, 2009), resulting in maintenance of the mitochondrial transmembrane potential (ΔΨ_m_) and prevention of release of pro-apoptotic proteins.

Mature neutrophils contain a low number of mitochondria that may have a role restricted to apoptosis (Maianski et al., 2004). Thus, mitochondrial respiration in mature neutrophils is low and mitochondria generate only small amounts of ATP by oxidative phosphorylation (Maianski et al., 2004). The mitochondrial poison cyanide does not affect neutrophil function. Nevertheless, neutrophil mitochondria maintain a transmembrane potential, forms a complex network that plays a role in chemotaxis, phagocytosis, and triggering apoptosis (Fossati et al., 2003). Mitochondria contains pro-apoptotic proteins cytochrome c, second mitochondria-derived activator of caspases (Smac)/DIABLO (direct IAP-binding protein with low pl), apoptosis-inducing factor (AIF), and endonuclease G (Saelens et al., 2004). Loss of ΔΨ_m_ evokes release of these proteins. Cytochrome c and Smac appear to be required for optimal caspase-3 activation (Altznauer et al., 2004). Loss of ΔΨ_m_ precedes development of apoptotic morphology in neutrophils undergoing constitutive (Maianski et al., 2004) and induced apoptosis.

### Role of reactive oxygen species and redox balance

Neutrophils generate high amounts of ROS by NADPH oxidase in response to soluble stimuli as well as following phagocytosis of bacteria in order to destroy invading pathogens (Nauseef, 2007). High amounts ROS can inflict damage to the surrounding cell and evoke necrosis. ROS also function as intracellular signaling molecules. The intrinsic or mitochondrial pathway of apoptosis is likely initiated through ROS generation (Kasahara et al., 1997; Maianski et al., 2004; Xu et al., 2010), though the source(s) of ROS in aging non-activated neutrophils is still unknown. Ligation of the cell surface death receptors, TNF, Fas, or TRAIL (TNF-related apoptosis-inducing ligand) receptors triggers the formation of the death-inducing signaling complex (DISC), which through downstream adaptor proteins, such as Fas-associated death domain (FADD) leads to activation of NADPH oxidase and cleavage of caspase-8 (Green, 2000). Likewise, phagocytosis of opsonized microorganisms through complement and/or Fcγ receptors evokes ROS-mediated activation of caspase-8 and subsequently caspase-3, leading to neutrophil apoptosis (Watson et al., 1996; Perskvist et al., 2002; DeLeo, 2004). Consistent with these findings, patients with chronic granulomatous diseases that lacks functional NADPH oxidase exhibit increased neutrophil viability (Fadeel et al., 1998) and reduced neutrophil apoptosis following ingestion of bacteria (Coxon et al., 1996). The effects of ROS are balanced by neutrophil antioxidant defenses, including catalase, superoxide dismutase, and glutathione. These proteins become depleted during *ex vivo* culture of neutrophils parallel with development of apoptotic morphology (Watson, 2002; Melley et al., 2005). Loss of GSH by chloramines or GSH depletion during activation of the respiratory burst predisposes to apoptosis (Melley et al., 2005).

### Regulation of apoptosis by granular and nuclear proteins

Certain granular and nuclear proteins have also been implicated in the modulation of the cell death program. For example, cathepsin D is released from the azurophilic granules during apoptosis and may contribute to activation of caspase-3 through processing of caspase-8 and Bid (Conus et al., 2008). Consistently, pharmacological or genetic inhibition of cathepsin D results in delayed neutrophil apoptosis. Mature neutrophils constitutively express the cyclin-dependent kinases CDK1, CDK2, and CDK5 (Rosales et al., 2004; Rossi et al., 2006). Culture of neutrophils with R-roscovitine, a non-selective inhibitor of cyclin-dependent kinases enhances apoptosis likely through down-regulation of Mcl-1 expression (Rossi et al., 2006; Leitch et al., 2010). Unlike in other cell types, proliferating cell nuclear antigen (PCNA) is expressed in the cytoplasm of mature neutrophils and is bound to pro-caspases, resulting in suppression of neutrophil apoptosis (Witko-Sarsat et al., 2010). Conversely, decreased cytoplasmic PCNA expression resulted in augmented neutrophil apoptosis. In contrast, during constitutive apoptosis, another nuclear protein myeloid nuclear differentiation antigen (MNDA) is cleaved by caspases and accumulates in the cytoplasm, where it promotes proteasomal degradation of Mcl-1 and subsequently collapse of mitochondrial transmembrane potential (Fotouhi-Ardakani et al., 2010). Bacterial constituents and platelet-activating factor prevent cytoplasmic MNDA accumulation parallel with preservation of Mcl-1 and suppression of apoptosis (Fotouhi-Ardakani et al., 2010).

### Suppression of apoptosis: Intracellular survival pathways

Although apoptosis is a default fate of neutrophils, in the inflammatory microenvironment, neutrophils are likely exposed to various pro-survival signals, including granulocyte macrophage colony stimulating factor (GM-CSF) (Colotta et al., 1992; Lee et al., 1993), leukotriene B_4_ (Lee et al., 1999), C5a (Lee et al., 1993), the acute-phase reactants C-reactive protein (Khreiss et al., 2002), and serum amyloid A (El Kebir et al., 2007; Christenson et al., 2008) as well as bacterial constituents LPS (Colotta et al., 1992; Lee et al., 1993) and bacterial DNA (József et al., 2004). Multiple kinase pathways are involved in determining the fate of neutrophils. For example, GM-CSF activates the Jak2/STAT and phosphoinositide-3-kinase (PI3K)/Akt pathways, leading to preservation of Mcl-1 expression and retardation of apoptosis (Klein et al., 2000; Epling-Burnette et al., 2001). PI3K generates PtdIns(3,4,5)P3, which also influences NF-κB and cAMP-response-element-binding protein (CREB) and thus may generate additional pro-survival signals (Ward et al., 2004). Many inflammatory mediators also activate the MAPK/ERK pathway that, in turn, inhibits the intrinsic pathway of apoptosis (Filep and El Kebir, 2010; Geering and Simon, 2011). ERK 1/2 and Akt phosphorylate Bad and Bax, leading to dissociation of phosphorylated Bad and Bax from the anti-apoptotic protein Mcl-1 (Akgul et al., 2001; El Kebir et al., 2007). Concomitant activation of Akt and ERK appears to be required for suppression of neutrophil apoptosis, and transient activation of Akt without ERK activation may not be sufficient to delay the death program. Contradictory results have been reported for p38 MAPK; its action on neutrophil survival may be stimulus and/or context-specific (reviewed in Filep and El Kebir, 2010). For example, pro-survival function of p38 MAPK may include phosphorylation, and therefore inactivation of caspase-3 and caspase-8 (Alvarado-Kristensson et al., 2003). In other studies, constitutive or TNF-induced neutrophil apoptosis was found to be associated with phosphorylation of p38 MAPK (Khreiss et al., 2002; El Kebir et al., 2007). Activation of p38 MAPK by sodium salicylate is associated with reduced Mcl-1 expression and acceleration of apoptotic cell death (Derouet et al., 2006).

## Neutrophil β_2_ Integrins Modulate Life and Death decisions

### β_2_ integrin activation and function

The β_2_ integrin (αβ) family consists of LFA-1 (leukocyte function-associated antigen 1, CD11a/CD18), Mac-1 (CD11b/CD18, α_M_β_2_ integrin, ITAM antigen), p150,95 (CD11c/CD18, α_X_β_2_ integrin, ITAX antigen), and α_d_β_2_ (CD11d/CD18, ITAD antigen). The β_2_ integrins are in an inactive (low affinity) conformation on circulating leukocytes. Leukocyte agonists trigger inside-out signaling that through activation of Rap1 (reviewed in Evans et al., 2009) induces conformational changes that reflect the intermediate and high affinity states of Mac-1 (Xiong et al., 2001; Luo et al., 2007). Ligand occupancy, but not integrin clustering promotes switchblade-like extension of the Mac-1 extracellular domain and separation of the α_M_ and β_2_ subunit cytoplasmic tails, structural hallmarks of integrin activation (Lefort et al., 2009). These lead to enhanced affinity for binding their ligand and/or regulation of avidity. Integrin activation is a complex and tightly regulated process which involves displacement of inhibitory proteins from the integrin cytoplasmic tail followed by targeting integrin activators or activator complexes, such as talin, kindlins, integrin-linked kinase, and migfilin (Kim et al., 2011). β_2_ integrins contribute to diverse neutrophil functions critical for innate immunity. Activated β_2_ integrins mediate leukocyte adhesion and transmigration across the endothelium through interactions with ICAM-1 on the activated endothelial cells (Ley et al., 2007; Abram and Lowell, 2009). Mac-1 also mediates other neutrophil adhesion-dependent functions, including binding to fibrinogen (Pluskota et al., 2004), immune complexes, and platelets (through Gp1b) (Mayadas and Cullere, 2005) and suppression of T cell proliferation (Pillay et al., 2012). Mac-1 and CD11c/CD18 are specific receptors for complement iC3b and mediate efficient phagocytosis of complement-opsonized targets, though they can also recognize many pathogens directly (Ross, 2000). Mac-1 functionally cooperates with other surface receptors, including TNF receptor, FcγRs, Toll-like receptor 2 (TLR2), and CD14 (Ehlers, 2000; Ross, 2000; Kobayashi et al., 2002; Salamone et al., 2004).

### Mac-1-mediated pro-survival signaling

**Figure 1:**
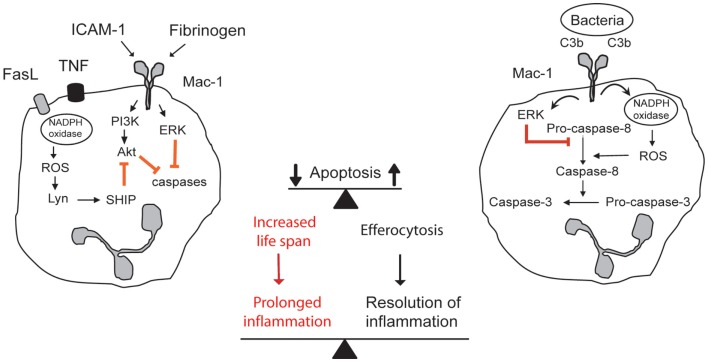
**Contrasting outside-in signals through Mac-1 modulates survival and death decision in neutrophils**. Aging neutrophils undergo constitutive apoptosis triggered by collapse of mitochondrial function. Engagement of Mac-1 with its ligands ICAM-1, fibrinogen, or MPO delays apoptosis by generating survival cues through activation of the PI3K/Akt and MEK/ERK pathways. Additional stimulation of death receptors with TNF or FasL evokes release of ROS, which through activation of lyn and SHIP leads to inhibition of Akt. Mac-1-mediated phagocytosis of opsonized bacteria evokes ROS production by NADPH oxidase, leading to ROS-mediated activation of caspase-8 and inhibition of ERK, and acceleration of the cell death program. Apoptotic neutrophils are recognized and phagocytosed by macrophages (efferocytosis), thus contributing to inflammatory resolution. Extended neutrophil longevity contributes to aggravation and prolongation of the inflammatory response.

Outside-in signaling through Mac-1 could generate contrasting cues for neutrophils in a context-dependent fashion (Figure 1). Transendothelial migration of neutrophils (Watson et al., 1997; Yan et al., 2004) or neutrophil adherence to Mac-1 ligands, ICAM-, fibrinogen, and plasminogen, prolongs their lifespan by delaying apoptosis (Table 1). Binding of ICAM-1 to Mac-1 induces activation of the PI3k/Akt survival pathway (Whitlock et al., 2000). Fibrinogen-mediated suppression of neutrophil apoptosis also depends on Akt in addition to activation of NF-κB and the MAPK/ERK pathway (Rubel et al., 2003). Crosslinking activated Mac-1 with anti-Mac-1 antibody (Fab fragments) or clustering inactive Mac-1 in neutrophils in suspension signals survival cues through activation of Akt and ERK (Whitlock et al., 2000). Soluble fibrinogen activates neutrophils, as assessed by upregulation of Mac-1 expression and elevation of intracellular calcium concentration (Rubel et al., 2003; Pluskota et al., 2008), indicating that Mac-1-mediated adhesion *per se* is not a prerequisite for generation of survival signals. Engagement of both Mac-1 subunits with these ligands is a prerequisite for induction of pro-survival signals (Pluskota et al., 2008). Consistently, angiostatin, derived from plasminogen and neutrophil inhibitory factor (NIF), which interact primarily with the α_M_ subunit do not trigger phosphorylation of ERK 1/2 and Akt and do not rescue neutrophils from constitutive apoptosis (Pluskota et al., 2008).

**Table 1:** **Selective regulation of neutrophil apoptosis by different ligands of Mac-1**.

Ligand	Action(s)	Effect on apoptosis	Reference
ICAM-1	Mediates PMN adhesion and transmigration	Suppresses apoptosis	Watson et al. (1997) Whitlock et al. (2000) Yan et al. (2004)
Fibrinogen	Precursor of fibrin initiates coagulation	Suppresses apoptosis	Rubel et al. (2001) Rubel et al. (2003) Pluskota et al. (2004, 2008)
Plasminogen	Precursor of plasmin initiates fibrinolysis	Suppresses apoptosis	Pluskota et al. (2008)
Angiostatin	Inhibits angiogenesis	No effect	Pluskota et al. (2008)
Myeloperoxidase	Bacterial killing upregulates Mac-1 expression induces MPO release	Suppresses apoptosis	El Kebir et al. (2008) Lau et al. (2005)
Heparin	Anticoagulant		
Immobilized	mediates leukocyte adhesion	Induces apoptosis	Diamond et al. (1995)
Soluble	inhibits binding of fibrinogen	Not known	Manaster et al. (1996)
Soluble, low molecular weight		No effect	Peters et al. (1999) Erduran et al. (1999) Brown et al. (2012)
Opsonized bacteria	Phagocytosis destruction of bacteria	Induces apoptosis	Coxon et al. (1996) Watson et al. (1999) Arroyo et al. (2002) Zhang et al. (2003) Perskvist et al. (2002) DeLeo (2004)

*MPO, myeloperoxidase; PMN, polymorphonuclear granulocytes*.

Heparin also binds to Mac-1. Immobilized heparin can mediate leukocyte adhesion (Diamond et al., 1995), whereas unfractionated soluble heparin was reported to inhibit binding of fibrinogen and complement iC3b to Mac-1 (Peters et al., 1999). Inconsistent reports have been published on the effect of heparin on neutrophil lifespan. Unfractionated heparin was reported to induce apoptosis (Manaster et al., 1996), whereas low molecular weight heparin did not affect neutrophil apoptosis (Erduran et al., 1999; Brown et al., 2012).

In the presence of TNF or anti-Fas activating antibody, crosslinking Mac-1 with activating antibodies promotes neutrophil apoptosis (Whitlock et al., 2000). Activation of TNF receptor or Fas results in NADPH oxidase-mediated ROS generation and extracellular ROS release, leading to activation of SHIP [Src-homology 2(SH2)-containing inositol 5-phosphatase], which hydrolyzes PI3K products through the Src kinase Lyn (Gardai et al., 2002). This would lead to decreased Akt phosphorylation. Although Lyn was reported to exert anti-apoptotic actions in granulocytes (Daigle et al., 2002), exogenous H_2_O_2_ can activate Lyn independently of adhesion (Yan and Berton, 1996). ROS, most likely H_2_O_2_ that was released extracellularly might diffuse back into cells to suppress the PI3K/Akt survival signal (Zhu et al., 2006; Xu et al., 2010). Whether ROS affect signaling pathways leading to activation of PI3K or through activation of PTEN (phosphatase and tensin homolog) is still uncertain. PTEN converts phosphatidylinositol 3,4,5-triphosphate to phosphatidylinositol-4,5-diphosphate, thus preventing activation of Akt. PTEN-null neutrophils live longer than wildtype neutrophils (Zhu et al., 2006). In contrast, crosslinking Mac-1 with clustering non-activating antibodies or recombinant ICAM-1 results in partial attenuation of Fas-triggered apoptosis through sustained ERK activation and elevation in reduced glutathione (GSH) levels (Watson et al., 1997; Whitlock et al., 2000). The biological significance of differences in neutrophil responses to Mac-1 activating versus clustering antibodies remains to be investigated. TNF promotion of neutrophil apoptosis evoked by immune complexes or zymosan partially depends on Mac-1 (Salamone et al., 2004), suggesting functional cooperation of Mac-1 with Fcγ or zymosan receptors (Ehlers, 2000; Ross, 2000).

While most studies investigated Mac-1 signaling, ligation of LFA-1 may also generates contrasting cues for neutrophils. LFA-1-deficient mice exhibit neutrophilia and enhanced resistance to *L. monocytogenes* without changes in spontaneous apoptosis (Miyamoto et al., 2003). Similar to Mac-1, ligation of LFA-1 during transendothelial migration suppresses caspase-3 activation and thus delays apoptosis in neutrophils (Yan et al., 2004). In contrast, activation of ICAM-3 with a monoclonal antibody that recognizes an ICAM-3 epitope that binds its ligand LFA-1 was found to induce apoptosis (Kessel et al., 2006).

### Mac-1-mediated acceleration of apoptosis

Phagocytosis of opsonized bacteria or other targets accelerates apoptosis in neutrophils, also referred to as phagocytosis-induced cell death or PICD (Coxon et al., 1996; Watson et al., 1999; Perskvist et al., 2002; DeLeo, 2004). Mac-1-mediated phagocytosis evokes NADPH-dependent ROS generation within the phagolysosomes (Karlsson and Dahlgren, 2002) and is thought to contribute to killing bacteria (Nauseef, 2007) as well as to triggering PICD (Watson et al., 1996; Zhang et al., 2003). Consistently, patients with chronic granulomatous disease, who have low level of NADPH oxidase due to genetic mutations in gp91^phox^ or other phox genes, suffer from recurrent infections (Heyworth et al., 2003) and their neutrophils do not undergo PICD (Coxon et al., 1996).

Mac-1-mediated phagocytosis evokes generation of NADPH oxidase-derived ROS, which, in turn, leads to activation of caspase-8 and subsequently caspase-3 (Arroyo et al., 2002; Zhang et al., 2003). ROS, most likely hydroxyl radicals and H_2_O_2_ (Watson et al., 1996; Perskvist et al., 2002) released within phagosomes might leak and trigger PICD. Superoxide released extracellularly is rapidly dismutated to H_2_O_2_, which might diffuse back to the cell to affect the redox status and signaling pathways. As caspase-8 activation is a signature of death receptor-mediated apoptosis, Mac-1 has been suggested to function as a death receptor, even though it does not contain a recognized death effector domain (Mayadas and Cullere, 2005). It should be noted that many stimuli that generate varying amounts of NADPH oxidase-derived ROS do not trigger neutrophil death. For example, GM-CSF enhances ROS production upon yeast phagocytosis, but inhibits PICD (Zhang et al., 2003). Thus, the amount, nature, and intra- or extracellular release of NADPH oxidase-derived ROS would likely determine their pro-apoptosis potential. PICD occurs despite phagocytosis-induced activation of the MAPK/ERK pathway (Zhang et al., 2003), indicating that ROS triggered pro-apoptosis signals effectively override survival cues. In contrast, GM-CSF evokes a more robust ERK phosphorylation in phagocytosing neutrophils, leading to generation of strong competing survival signals that shift the life-death balance toward survival (Zhang et al., 2003).

### Myeloperoxidase: A ligand for Mac-1 and survival signal for neutrophils

An unexpected ligand for Mac-1 is myeloperoxidase (MPO), the most abundant granule enzyme in neutrophils (Schultz and Kaminker, 1962; Borregaard and Cowland, 1997). MPO, MPO-generated reactive oxidants, hypochlorous acid (HOCl) in particular and diffusible radical species have been implicated in the elimination of microbes (Klebanoff, 2005; Nauseef, 2007; Davies et al., 2008) as well as in inflicting tissue damage (Klebanoff, 2005; Winterbourn, 2008; Arnhold and Flemming, 2010). Non-activated neutrophils bind to MPO-coated surfaces (Johansson et al., 1997) or “free” circulating MPO through Mac-1 (Lau et al., 2005). Increased MPO association with neutrophil membrane was detected in blood from patients with inflammatory diseases, including sepsis, ischemia-reperfusion, or acute coronary syndromes compared with healthy controls (Lau et al., 2005). Membrane-bound MPO correlates with plasma MPO levels, indicating up-regulation of MPO export toward the plasma membrane as well as a potential for binding of free MPO to the neutrophil surface.

**Figure 2:**
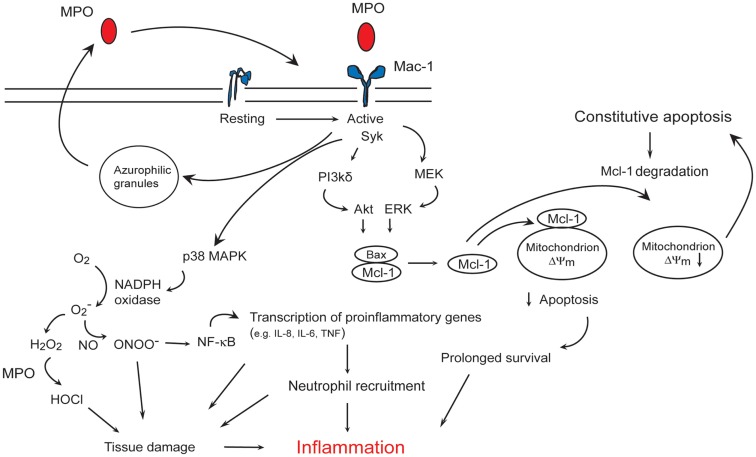
**Myeloperoxidase (MPO)-Mac-1 self-amplifying circuit promotes neutrophils survival and inflammation**. MPO binding to Mac-1 results in p38 MAPK-mediated NADPH oxidase, and PI3K/Akt and MEK/ERK-mediated preservation of the anti-apoptotic protein Mcl-1, leading to suppression of apoptosis. MPO also triggers MPO release from the azurophilic granules, and upregulates Mac-1 expression, thereby forming an autocrine/paracrine self-amplifying circuit. MPO catalyzed formation of HOCl, activation of NF-κB-regulated transcription of pro-inflammatory genes, recruitment of neutrophils, and prolongation of neutrophil longevity contribute to tissue damage and inflammation.

MPO binding to Mac-1 on human neutrophils leads to increased tyrosine phosphorylation (Lau et al., 2005), phosphorylation of p38 MAPK (Lau et al., 2005; El Kebir et al., 2008), ERK 1/2 and PI3K (El Kebir et al., 2008), and activation of NF-κB (Lau et al., 2005). Activation of p38 MAPK induces phosphorylation of p47^phox^, the cytoplasmic regulatory subunit of NADPH oxidase (Babior, 2004), leading to superoxide formation (Lau et al., 2005), and regulates NF-κB-mediated transcription of genes involved in the acute inflammatory response (Park et al., 2003). Intriguingly, MPO also upregulates surface expression of Mac-1 on neutrophils (Lau et al., 2005; El Kebir et al., 2009) through yet unidentified molecular mechanisms. MPO binding to Mac-1 induces release of elastase and MPO from the azurophilic granules (Lau et al., 2005). These findings are consistent with the central role of Mac-1-mediated outside-in signaling in degranulation (Harris et al., 2000), and imply an autocrine/paracrine mechanism for amplifying neutrophil responses to MPO (Figure 2) (El Kebir et al., 2008, 2009).

MPO, independent of its catalytic activity, rescues neutrophils from constitutive apoptosis through simultaneous activation of ERK 1/2 and Akt, Mcl-1 accumulation and suppression of the mitochondrial pathway of apoptosis (El Kebir et al., 2008). Consistently, MPO prevents cytochrome c release from the mitochondria and subsequent activation of caspase-3 (El Kebir et al., 2008). MPO-induced phosphorylation of p38 MAPK does not generate a survival signal, for pharmacological blockade of p38 MAPK retards neutrophil apoptosis both in the absence and presence of MPO (El Kebir et al., 2008). In contrast to these findings, MPO was found to mediate apoptosis in HL60 leukemia cells (Wagner et al., 2000; Kanayama and Miyamoto, 2007). While the involvement of Mac-1 in the pro-apoptosis action of MPO has not been elucidated, Mac-1 may play opposing roles in determining the fate of primary and leukemia cells likely by shifting the balance of pro-survival and pro-apoptosis cues. It is intriguing that MPO can prolong the lifespan of neutrophils, the predominant source of this enzyme and that function-blocking monoclonal anti-Mac-1 antibodies can almost completely prevent MPO-induced activation (Lau et al., 2005; El Kebir et al., 2008) and suppression of constitutive apoptosis of human neutrophils *in vitro* (El Kebir et al., 2008).

### Myeloperoxidase prolongs neutrophil lifespan and delays resolution of inflammation

Acute elevation of plasma MPO levels to levels similar to those detected in patients with inflammatory vascular diseases (Brennan et al., 2001; Baldus et al., 2003) results in prolongation of the lifespan of rat neutrophils through suppression of apoptosis as assayed *ex vivo* (El Kebir et al., 2008). MPO also suppresses neutrophil apoptosis in a mouse model of carrageenan-induced lung injury and delays spontaneous self-resolution of pulmonary inflammation (El Kebir et al., 2008). Thus, combined administration of carrageenan and MPO evokes persisting lung injury/inflammation with few airway neutrophil exhibiting signs of apoptosis even 5 days post-injection, when pulmonary inflammation is almost completely resolved in the lungs of carrageenan-injected mice. The effects of MPO closely resemble those of zVAD-fmk, a pan-caspase inhibitor, which aggravates and prolongs carrageenan-elicited acute pleurisy (Rossi et al., 2006) and lung inflammation (El Kebir et al., 2008). MPO-deficient mice exhibit lower pulmonary bacterial colonization, reduced lung injury, and greater survival following intraperitoneal injection of *Escherichia coli* compared with wild type mice (Brovkovych et al., 2008). MPO deficiency also reduces ischemia/reperfusion-induced renal dysfunction and neutrophil accumulation in mice, but fails to abrogate apoptosis during early phases of reperfusion (Matthijsen et al., 2007). Intriguingly, MPO-deficient mice exhibit elevated baseline pulmonary inducible NO synthase expression and NO production that may partially compensate for the lack of HOCl-mediated bacterial killing (Brovkovych et al., 2008). The mechanism(s) responsible for upregulation of inducible NO synthase as well as the impact of enhanced NO production on the resolution of lung inflammation remains to be explored. Absence of MPO-derived oxidant production during *E. coli* septicemia in MPO-null mice is consistent with reduced lung injury and mortality. Further studies are required to investigate whether MPO deficiency could affect the lifespan of emigrated or circulating neutrophils, and whether changes in neutrophil longevity could contribute to protection against lung injury in this model of sepsis.

## Targeting Mac-1 Signaling for Therapeutic Induction of Neutrophil Apoptosis

Accumulating experimental and clinical data suggest a causal relationship between neutrophil apoptosis and outcome of inflammation. Apoptosis of emigrated neutrophils has multiple pro-resolution actions. It renders neutrophils unresponsive to agonists and apoptotic neutrophils stop producing and releasing pro-inflammatory mediators. Apoptotic leukocytes sequester cytokines (Ariel et al., 2006; Ren et al., 2008) and phagocytosis of apoptotic cells induces macrophage polarization from a pro-inflammatory to a pro-resolution phenotype (Fadok et al., 1998; Ariel and Serhan, 2012; Sica and Mantovani, 2012). Injection of large number of apoptotic neutrophils protects mice against LPS-induced shock (Ren et al., 2008). Recent studies identified several classes of molecules for therapeutic induction of apoptosis in neutrophils for enhancing the resolution of inflammation.

### Lipoxins inhibit MPO signaling through Mac-1 and redirects neutrophils to apoptosis

The pivotal role of MPO in host defense and tissue injury makes it an attractive target for drug development. Indeed, while a number of promising compounds have been developed to inhibit the enzymatic activities of MPO, only limited information is available on their mechanisms of MPO inhibition and biological activities (reviewed in Malle et al., 2007). Targeting MPO signaling through Mac-1 has emerged as an alternative approach to counter the non-enzymatic activities of MPO.

Lipoxin A_4_ (LXA_4_) and aspirin-triggered 15-epi-LXA_4_ are typically generated by transcellular biosynthesis at sites of inflammation (Serhan et al., 2008; Serhan, 2011). In the aspirin-triggered pathway, acetylation of cyclooxygenase at Ser^530^ by aspirin (Clària and Serhan, 1995) or S-nitrosylation at Cys^526^ by atorvastatin (Birnbaum et al., 2006) catalyzes the conversion of arachidonate to 15R-HETE that can be converted by neutrophils and other cells to 15-epi-LXA_4_ and 15-epi-LXB_4_. LXA_4_ and 15-epi-LXA_4_ possess potent anti-inflammatory and pro-resolution actions predominantly through affecting the function of leukocytes. Lipoxins stimulate recruitment of monocytes and inhibit neutrophil trafficking and accumulation in inflamed tissues (reviewed in Serhan et al., 2007; Serhan et al., 2008). These actions are, in part, mediated through down-regulation of leukocyte Mac-1 expression (Fiore and Serhan, 1995; Filep et al., 1999). Accumulation of PGE_2_ at inflammatory sites induces a lipid mediator class switching from a predominantly 5-lipoxygenase activity to a 15-lipoxygenase activity generating LXA_4_ parallel with the resolution of inflammation (Levy et al., 2001) Thus, initiation of an inflammatory response would also activate subsequent pro-resolution mechanisms (Serhan and Savill, 2005).

**Figure 3:**
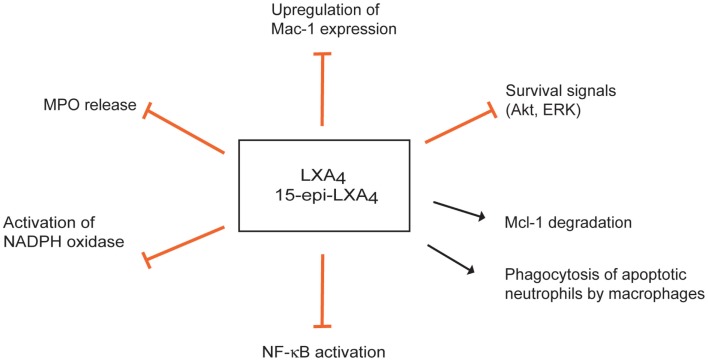
**Multipronged actions of lipoxins to inhibit the myeloperoxidase (MPO)-Mac-1 circuit**. Lipoxin A_4_ (LXA_4_) and aspirin-triggered 15-epi-LXA_4_ predominantly through FPR2/ALX attenuate MPO-stimulated degranulation, upregulation of surface expression of Mac-1, and superoxide formation, thus effectively interrupting this loop. LXA_4_ and15-epi-LXA_4_ redirect neutrophils to apoptosis by overriding the powerful survival signals from MPO through inducing loss of expression of Mcl-1 and aggravating mitochondrial dysfunction. Lipoxins also enhance phagocytosis of apoptotic neutrophils by macrophages.

Lipoxins exert multipronged actions to counter neutrophil responses to MPO. Down-regulation of Mac-1 expression on neutrophils and inhibition of neutrophil adhesion and transendothelial migration are important components of the anti-inflammatory activities of LXA_4_ and 15-epi-LXA_4_ (Serhan et al., 2008). 15-epi-LXA_4_ also prevents MPO-induced up-regulation of Mac-1 expression and MPO release, thereby interrupting MPO-mediated autocrine/paracrine loop for perpetuation of the inflammatory response (Figure 3) (El Kebir et al., 2009). 15-epi-LXA_4_ inhibition of NADPH oxidase-derived superoxide generation (Levy et al., 1999) and subsequent formation of ONOO^−^ (József et al., 2002) result in reduced NF-κB activation and transcription of pro-inflammatory cytokines, such as IL-8 (József et al., 2002).

Lipoxins themselves do not appear to interfere with the apoptotic machinery in neutrophils, whereas they can override the potent outside-in Mac-1-mediated survival signal and redirect neutrophils to apoptosis *in vitro* (El Kebir et al., 2009). 15-epi-LXA_4_ attenuates MPO-evoked ERK and Akt-mediated phosphorylation of the pro-apoptotic protein Bad and decreases Mcl-1 expression, critical events in enhancing neutrophil apoptosis. Non-phosphorylated Bad associates with Mcl-1 and prevents its anti-apoptotic actions (Reed, 2006). These would aggravate mitochondrial dysfunction, ultimately leading to caspase-3-mediated cell death (El Kebir et al., 2009; Wardle et al., 2011).

Treatment of mice with 15-epi-LXA_4_ at the peak of inflammation enhances resolution of carrageenan plus MPO-induced and *E. coli* septicemia-associated acute lung injury and improves the survival rate (El Kebir et al., 2009). 15-epi-LXA_4_ reduces pulmonary neutrophil accumulation with concomitant increases in the percentage of apoptotic neutrophils in the airways, facilitates recruitment of monocytes/macrophages and phagocytosis of apoptotic neutrophils and other cells (El Kebir et al., 2009), consistent with tissue repair (Godson et al., 2000; Mitchell et al., 2002). Furthermore, LXA_4_ released at sites of inflammation protects macrophages from apoptosis (Prieto et al., 2010). The beneficial actions of 15-epi-LXA_4_ can be prevented in the presence of a pan-caspase inhibitor, indicating the importance of neutrophil apoptosis in inflammatory resolution. Recent results indicate that aspirin or lovastatin reduction of acid aspiration-induced lung inflammation is, in part, mediated through stimulation of synthesis of 15-epi-LXA_4_ (Fukunaga et al., 2005; Planaguma et al., 2010). The direct effect of lovastatin on neutrophil apoptosis remains, however, to be investigated. Both aspirin and sodium salicylate promote neutrophil apoptosis and enhance their phagocytosis by macrophages in thioglycollate-induced peritonitis (Negrotto et al., 2006). A recent study reported that serum amyloid A acting through the formyl-peptide receptor 2/lipoxin receptor (FPR2/ALX) can overwhelm anti-inflammatory signaling by LXA_4_ to mediate exacerbation of glucocorticoid refractory lung inflammation in patients with chronic obstructive pulmonary disease (Bozinovski et al., 2012).

### Resolvin E1 promotes phagocytosis-induced neutrophil apoptosis

Resolvin E1 is synthesized from the ω-3 polyunsaturated fatty acid eicosapentaenoic acid during the resolution phase of acute inflammation with leukocyte 5-lipoxygenase playing a pivotal temporal role in the biosynthesis pathway (Serhan et al., 2000; Oh et al., 2011). RvE1 binds to the ChemR23 and (as a partial agonists/antagonist) the leukotriene B_4_ (LTB_4_) receptor BLT1 (Arita et al., 2005; Oh et al., 2011) and exhibits potent anti-inflammatory and pro-resolution activities. Thus, RvE1 inhibits neutrophil recruitment, facilitates efferocytosis (Serhan et al., 2002, 2008; Ohira et al., 2010; Oh et al., 2011; Serhan and Petasis, 2011), promotes mucosal surface clearance (Campbell et al., 2007), and induces generation of LXA_4_ (Haworth et al., 2008).These potent pro-resolution actions were also demonstrated in various experimental models, including peritonitis (Oh et al., 2011), polymicrobial sepsis (Schwab et al., 2007), and bacterial pneumonia (Seki et al., 2010) Moreover, studies on ChemR23-null mice demonstrated an endogenous anti-inflammatory role for ChemR23 (Cash et al., 2008).

Recent results indicate that RvE1 can modulate neutrophil apoptosis (El Kebir et al., 2012). While at low nanomolar concentrations, RvE1 *per se* does not affect the constitutive death program in neutrophils, it enhances Mac-1-mediated phagocytosis of complement-opsonized bacteria and yeast, leading to increased ROS generation by NADPH oxidase and subsequent activation of caspase-8 and caspase-3 (El Kebir et al., 2012). RvE1 also attenuates ERK and Akt-mediated survival cues generated by MPO and decreases Mcl-1 expression, thereby reinforcing the shift toward apoptosis (El Kebir et al., 2012). These actions of RvE1 are predominantly mediated via BLT1 *in vitro*, indicating that resolution mechanisms may also be activated via this type of LTB_4_ receptor. In contrast, RvE1 stimulation of phagocytosis of live *E. coli* and apoptotic neutrophils by macrophages leads to a macrophage phenotype switch without evoking apoptosis (Arita et al., 2005; Schwab et al., 2007; Oh et al., 2011). Since in macrophages RvE1 signals via ChemR23 (Ohira et al., 2010), RvE1 may exert different pro-resolution actions via distinct receptors, and concurrent activation of these circuits may be critical for optimal resolution. The neutrophil apoptosis-promoting action of RvE1 was also evident in experimental models of acute respiratory distress (ARDS) and pneumonia (El Kebir et al., 2012), in which MPO has been implicated in mediating lung injury. RvE1 administered at the peak of inflammation, promoted apoptosis in neutrophils *in situ*, enhanced recruitment of monocytes to the airways, and facilitated clearance of apoptotic neutrophils and other cells and tissue repair (El Kebir et al., 2012), consistent with the original properties defining RvE1 actions (Serhan et al., 2008). Efficient resolution of acute lung inflammation is intimately linked to apoptosis of neutrophils within the airways, as the pan-caspase inhibitor zVAD-fmk prevented RvE1-induced dramatic reduction in the number of infiltrating neutrophils (El Kebir et al., 2012) and aggravated lung injury likely due to persisting presence of neutrophils. Eicosapentaenoic acid is also a substrate for acetylated COX-2, which generates aspirin-triggered resolvins that shares anti-inflammatory actions of native resolvins (Spite and Serhan, 2010). These would raise the possibility that resolvin-triggered phagocytosis-induced neutrophil apoptosis could contribute to the beneficial actions of aspirin.

### Other approaches to induce neutrophil apoptosis *in vivo*

**Figure 4:**
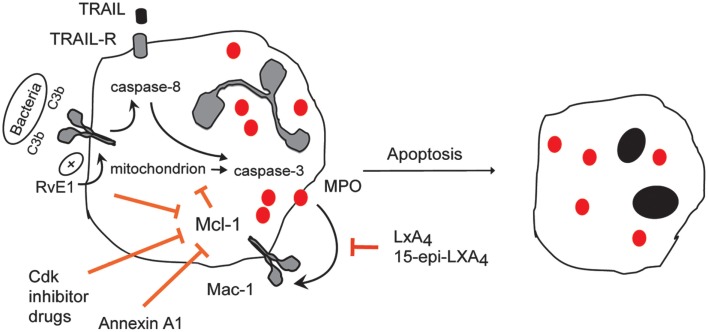
**Therapeutic induction of neutrophil apoptosis with demonstrated pro-resolution actions *in vivo***. Treatment of experimental animals with CDK inhibitor drugs (e.g., R-roscovitine), 15-epi-LXA_4_, RvE1, rTRAIL, or annexin A1 enhance the resolution of inflammation by promoting apoptosis of neutrophils that have emigrated into tissues in various models of inflammation. Roscovitine through yet unidentified mechanisms, 15-epi-LXA_4_ through attenuating MPO-triggered Mac-1-mediated survival signaling and annexin A1 through dampening intracellular survival signaling down-regulate expression of the key survival protein Mcl-1. RvE1 enhances phagocytosis-induced apoptosis, leading to activation of caspase-8 and suppression of Mcl-1.rTRAIL activates caspase-8.

Given the complexity of pathways involved in the regulation of neutrophil apoptosis, a number of agents have been identified that could shift the balance of survival and pro-apoptosis cues toward apoptosis. More importantly, some of these agents have been found to possess pro-resolution properties in diverse models of inflammation (see also Figure 4).

At the light of its pre-eminence as a key survival protein, Mcl-1 is an attractive target for therapeutic induction of apoptosis. The cyclin-dependent kinase inhibitor R-roscovitine (Seliciclib or CYC202) accelerates degradation of Mcl-1 and inhibits transcriptional activity in neutrophils by preventing cyclin-dependent kinase (CDK) 7 and 9-mediated phosphorylation of RNA polymerase II (Leitch et al., 2012), thereby inducing apoptosis in inflammatory cells *in vitro* (Rossi et al., 2006; Leitch et al., 2012). Consistently, treatment of with R-roscovitine enhances resolution of pleuritis and bleomycin-induced lung injury (Rossi et al., 2006) and pneumococcal meningitis in mice (Koedel et al., 2009), coinciding with increased numbers of apoptotic neutrophils in the airways and cerebrospinal fluid, respectively. R-roscovitine induces apoptosis in neutrophils from patients with cystic fibrosis, whereas the selective CFTR inhibitor CFTR_Inh172_ does not affect constitutive apoptosis in neutrophils from healthy volunteers (Moriceau et al., 2010). Suppressed neutrophil apoptosis in cystic fibrosis is likely not simply a consequence of chronic infection. Since cystic fibrosis is associated with enhanced formation of MPO-generated oxidants, it would be interesting to know whether MPO is one of the yet unidentified modulatory factors intrinsic to CF.

The pro-resolution mediator annexin A1 binds to FPR2/ALX, which is also a receptor for LXA_4_ and 15-epi-LXA_4_, and accelerates neutrophil apoptosis in various murine models of inflammation by decreasing survival signals and Mcl-1 expression (Solito et al., 2003; Perretti and D’Acquisto, 2009; Perretti, 2012). A recent study reported that annexin A1 in inflammatory exudates promotes active resolution and augments neutrophil apoptosis in LPS-induced pleurisy in mice (Vago et al., 2012). Intriguingly, annexin A1-deficient mice exhibit an exaggerated inflammatory response and reduced Mac-1 expression on neutrophils (Hannon et al., 2003). Whether the pro-resolution actions of annexin A1 also involve modulation of signaling through Mac-1 remain to be explored. The phosphodiesterase 4 inhibitor rolipram has also recently been found to promote neutrophil apoptosis and resolution of LPS-induced pleurisy in mice by decreasing PI3K/Akt survival signaling and Mcl-1 expression (Sousa et al., 2010).

Another possibility is induction of caspase activity, the major effectors of apoptosis. Although selective caspase activators are currently not available, recent studies demonstrated the pro-resolving action of the death receptor ligand TRAIL, which may function as a physiological brake to limit the inflammatory response (Leitch et al., 2011). Thus, TRAIL-deficiency in mice is associated with delayed neutrophil apoptosis and exaggerated inflammatory response (Falschlehner et al., 2009; McGrath et al., 2011). Conversely, rTRAIL was found to facilitate neutrophil apoptosis through inducing activation of caspase-8 both *in vitro* and *in vivo* (Renshaw et al., 2003; McGrath et al., 2011). Consistent with enhanced neutrophil apoptosis, treatment with rTRAIL accelerated resolution of LPS-induced lung injury and zymosan-induced peritonitis in TRAIL-deficient mice (McGrath et al., 2011). Up-regulation of TRAIL has also been implicated in mediating TLR4 signaling through IFN-β to promote neutrophil apoptosis and limiting lung inflammation in a mouse model of ARDS (Leu et al., 2011).

Targeting pro-survival pathways to promote resolution has also been investigated at the level of MAPK and NF-κB signaling. As discussed above, pharmacological blockade of ERK 1/2 and/or PI3K prevents GM-CSF or MPO-induced neutrophil survival *in vitro*. The efficacy of the MEK/ERK inhibitor PD98059 in the resolution of inflammation was also demonstrated in the carrageenan-induced pleurisy model in rats (Sawatzky et al., 2006). NF-κB blockers have potential anti-inflammatory actions (Gosh and Hayden, 2008) and may also affect resolution. An oligonucleotide decoy to NF-κB was found to enhance neutrophil apoptosis and phagocytosis by macrophages in a rat model of chronic inflammation (Maiuri et al., 2004). Increased apoptosis correlated with increases in p53 or Bax expression and decreases in Bcl-2 protein expression. Systemic injection of a cell-permeable form of IκBα (Tat-srIκBα chimera) reduced leukocyte trafficking into the pleural cavity and increased caspase-3 activity and apoptosis in emigrated cells (Blackwell et al., 2004). In contrast, the Tat-srIκBα chimera produced only marginal reductions in neutrophil migration and apoptosis if administered locally (Blackwell et al., 2004), indicating that the route and timing of administration may be critical for exerting beneficial actions. In another study, selective NF-κB inhibitors failed to affect neutrophil accumulation in the pleural cavity (Sousa et al., 2010).

## Conclusion

Intensive research during the past decade has revealed that inflammation does not terminate spontaneously; rather resolution is a tightly controlled active process. During the past decade, a number of novel mediators, including lipids, peptides, and proteins, and signaling circuits have been identified. Timely and efficient removal of migrated neutrophils requires these cells to undergo apoptosis. A growing body of evidence supports an important role for neutrophil apoptosis as a critical control point for the outcome of inflammation. Neutrophil surface receptors, including the adhesion molecule Mac-1 integrates opposing cues that modulate life and death decisions and therefore the outcome of inflammation. Outside-in signaling through Mac-1 is also a target for endogenous molecules, such as lipoxins, resolvin E1, and synthetic compounds to counter pro-survival and/or induce pro-apoptosis signals. Interfering with Mac-1 function may have two important benefits: inhibition of neutrophil trafficking into the inflamed site and acceleration of neutrophil clearance from inflamed tissues through the process of efferocytosis. Indeed, results from experimental models demonstrate that redirecting neutrophils to apoptosis and facilitating their clearance by macrophages are essential for enhancing resolution of acute inflammation. While clinical trials with these compounds remain distant, therapeutic induction of neutrophil apoptosis at the inflammatory site hold promise as a powerful pro-resolving intervention and may fulfill urgent, yet unmet clinical needs to prevent the deleterious consequences of inflammation.

## Conflict of Interest Statement

The authors declare that the research was conducted in the absence of any commercial or financial relationships that could be construed as a potential conflict of interest.
